# Supplementary material for: GDF15 is required for cold-induced thermogenesis and contributes to improved systemic metabolic health following loss of OPA1 in brown adipocytes
Source: eLife. 2023 Oct 11;12:e86452. doi: 10.7554/eLife.86452 (PMC10567111; doi:10.7554/eLife.86452)
Supplement: Figure 3—source data 1. — (A) Full immunoblot images for OPA1, PERK, and GAPDH in brown adipose tissue (BAT) (uncropped blots with the relevant bands labeled). (B) Full immunoblot images for pEif2a and Eif2a in BAT. (F) Full immunoblot images for UCP1 and β-actin in BAT. (I) Full immunoblot images for UCP1 and β-actin in inguinal white adipose tissue (iWAT). (J) Full immunoblot images for tyrosine hydroxylase (TH) and β-actin in iWAT. [file elife-86452-fig3-data1.zip › Fig. 3 - source data 1.pptx]

## Slide 1
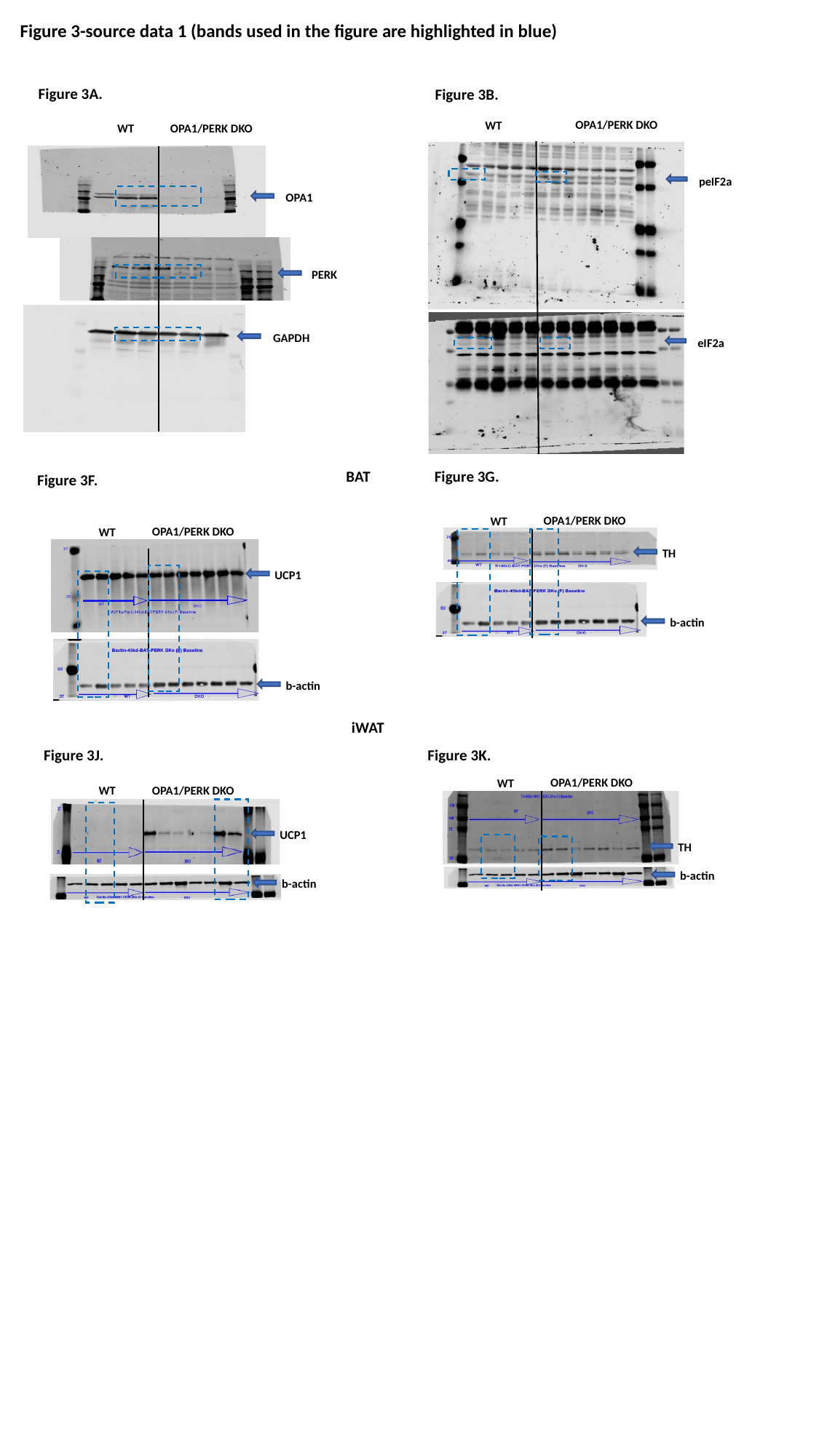

Figure 3-source data 1 (bands used in the figure are highlighted in blue)
Figure 3A.
Figure 3B.
OPA1/PERK DKO
WT
peIF2a
eIF2a
OPA1/PERK DKO
WT
OPA1
PERK
GAPDH
BAT
Figure 3G.
OPA1/PERK DKO
WT
TH
b-actin
Figure 3F.
OPA1/PERK DKO
WT
UCP1
b-actin
iWAT
Figure 3K.
Figure 3J.
OPA1/PERK DKO
WT
WT
OPA1/PERK DKO
UCP1
TH
b-actin
b-actin
