# Supplementary figures and images for: GDF15 is required for cold-induced thermogenesis and contributes to improved systemic metabolic health following loss of OPA1 in brown adipocytes
Source: eLife. 2023 Oct 11;12:e86452. doi: 10.7554/eLife.86452 (PMC10567111; doi:10.7554/eLife.86452)

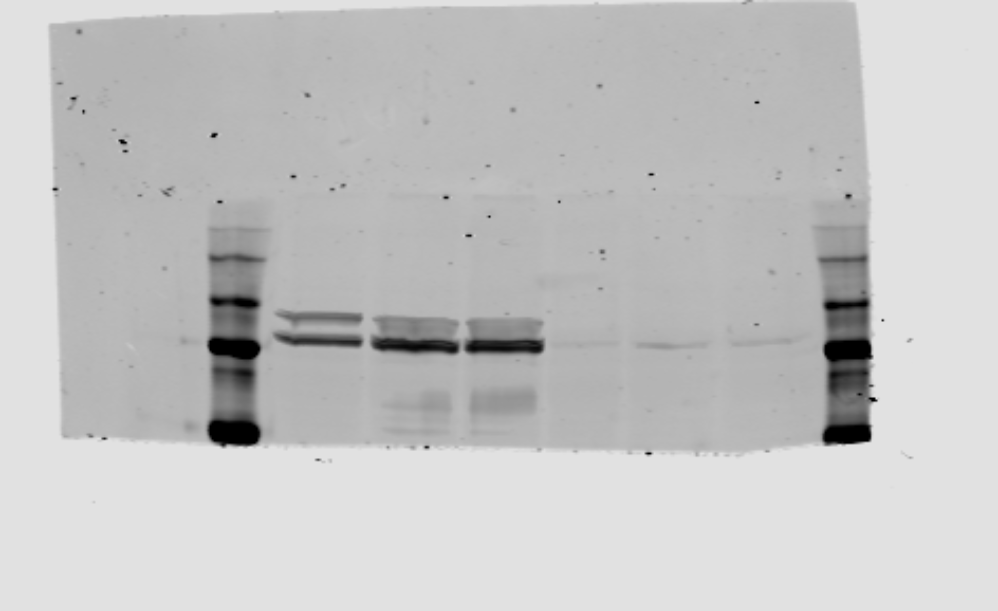

Supplement: Figure 3—source data 2. [file elife-86452-fig3-data2.zip › Figure 2 - source data 2.tif]

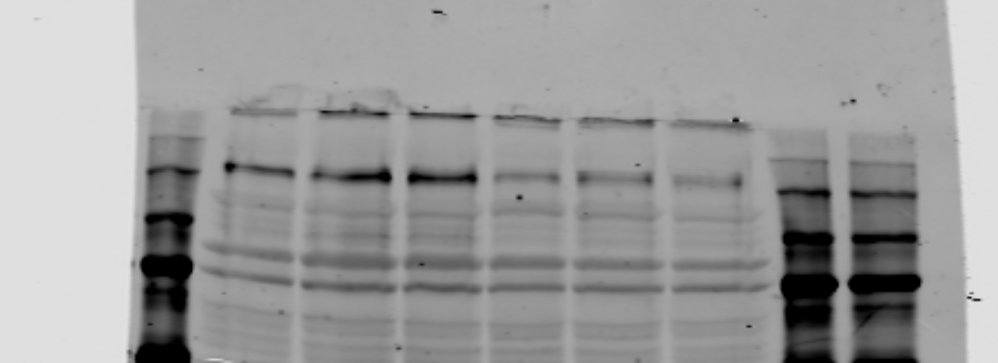

Supplement: Figure 3—source data 3. [file elife-86452-fig3-data3.zip › Figure 2 - Source data 3.tif]

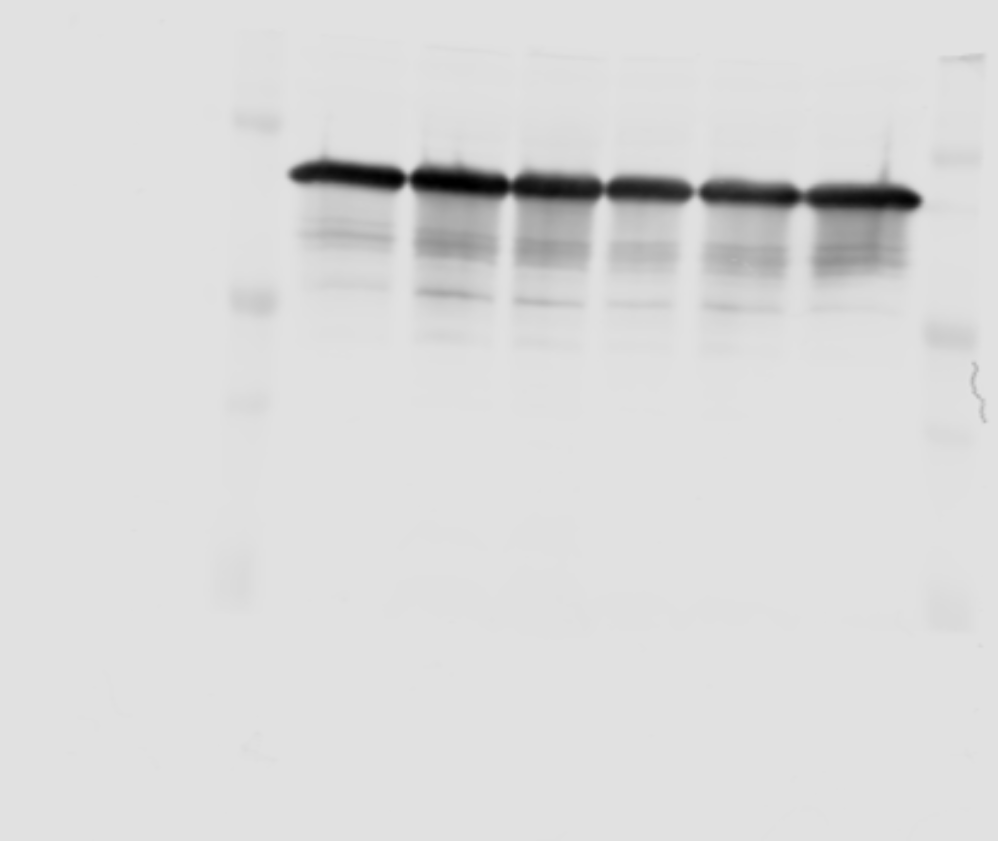

Supplement: Figure 3—source data 4. [file elife-86452-fig3-data4.zip › Figure 2 - source data 4.tif]

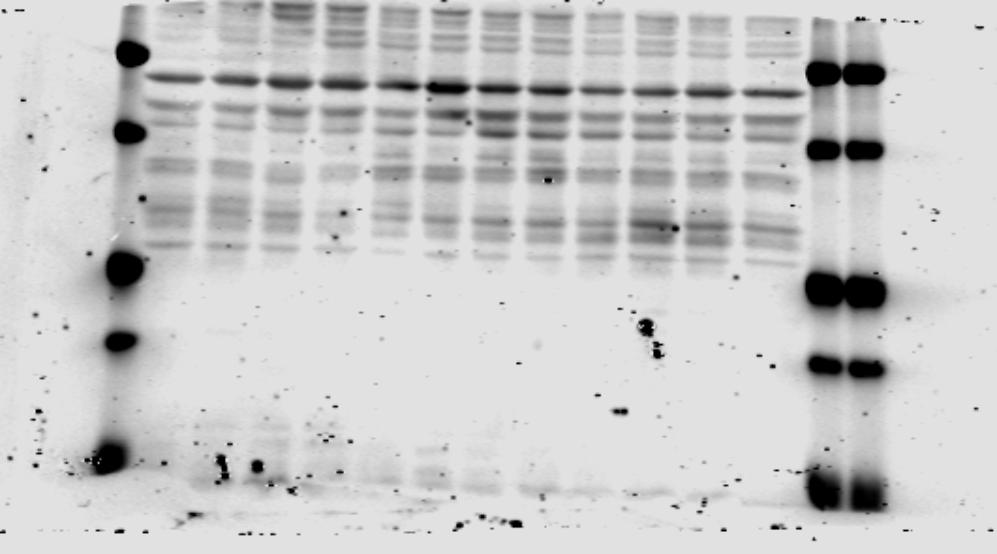

Supplement: Figure 3—source data 5. [file elife-86452-fig3-data5.zip › Figure 2 - source data 5.tif]

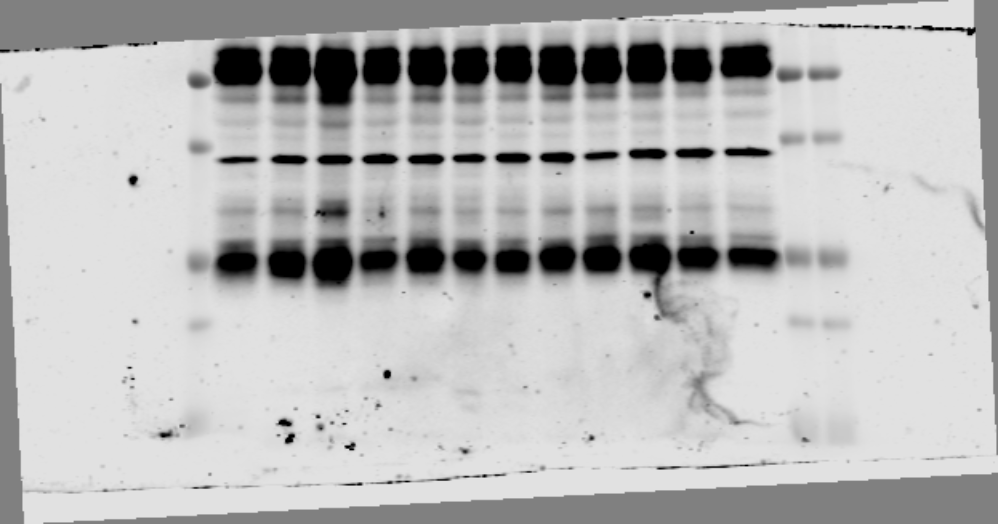

Supplement: Figure 3—source data 6. [file elife-86452-fig3-data6.zip › Figure 2 - source data 6.tif]

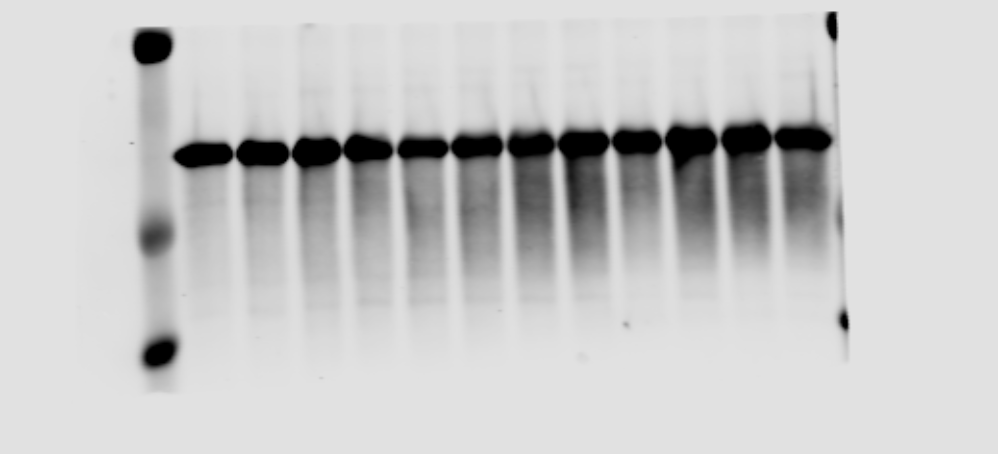

Supplement: Figure 3—source data 7. [file elife-86452-fig3-data7.zip › Fig.3 source data 7.tif]

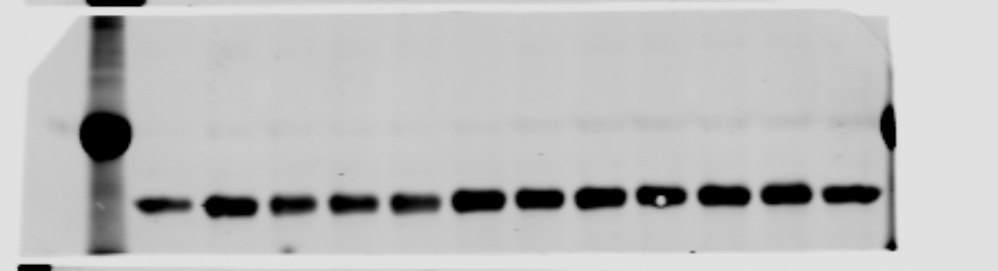

Supplement: Figure 3—source data 8. [file elife-86452-fig3-data8.zip › Fig.3 source data 8.tif]

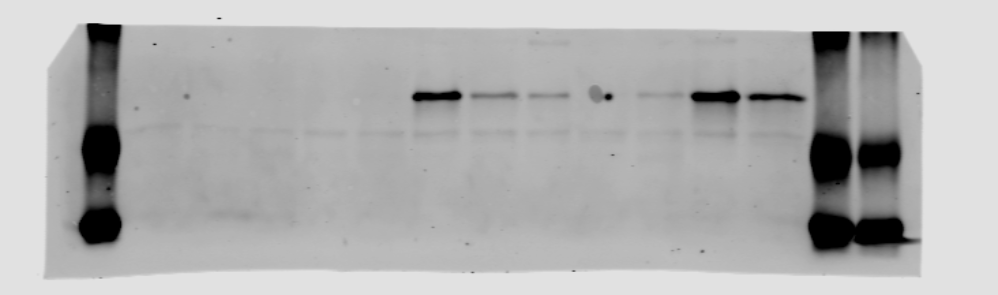

Supplement: Figure 3—source data 9. [file elife-86452-fig3-data9.zip › Fig.3 source data 9.tif]

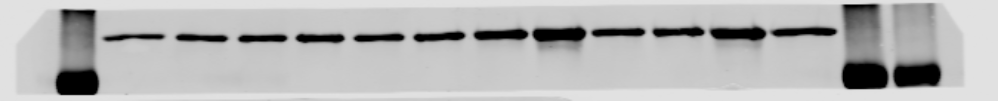

Supplement: Figure 3—source data 10. [file elife-86452-fig3-data10.zip › Fig.3 source data 10.tif]

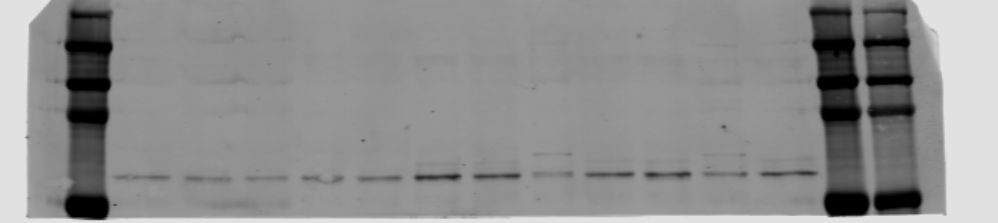

Supplement: Figure 3—source data 11. [file elife-86452-fig3-data11.zip › Fig.3 source data 11.tif]

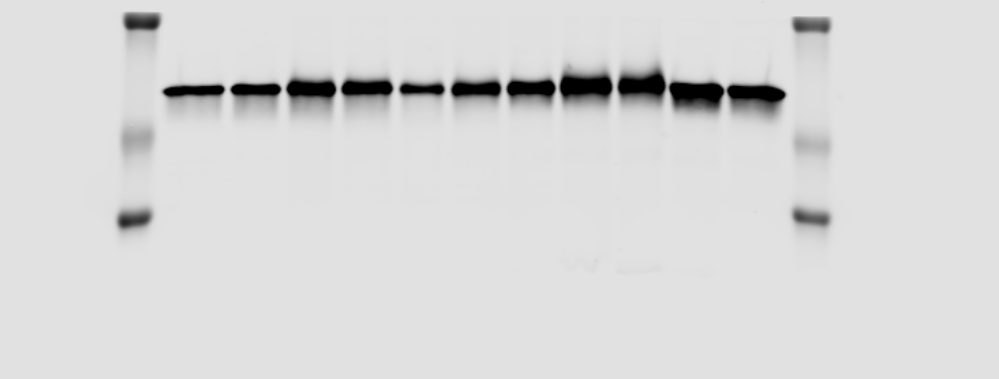

Supplement: Figure 5—source data 2. [file elife-86452-fig5-data2.zip › Fig.5 source data 2.tif]

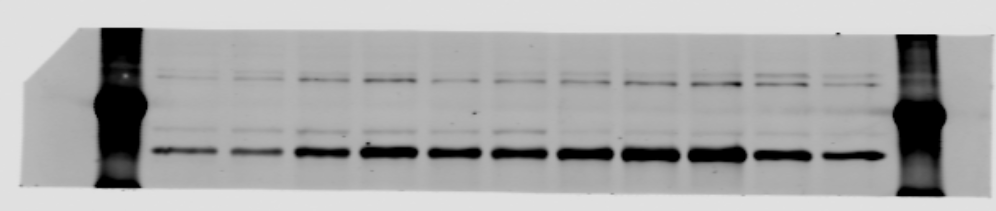

Supplement: Figure 5—source data 3. [file elife-86452-fig5-data3.zip › Fig.5 source data 3.tif]

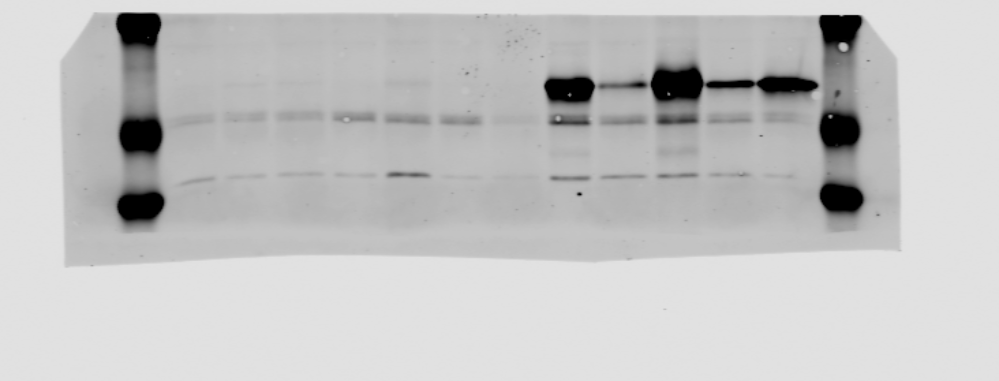

Supplement: Figure 5—source data 4. [file elife-86452-fig5-data4.zip › Fig.5 source data 4.tif]

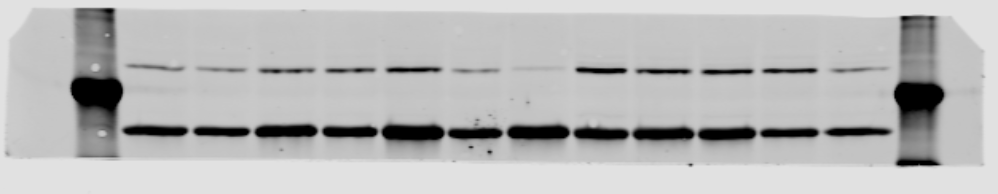

Supplement: Figure 5—source data 5. [file elife-86452-fig5-data5.zip › Fig.5 source data 5.tif]

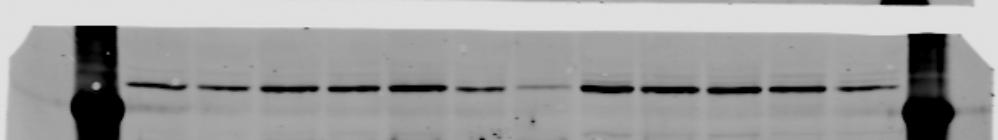

Supplement: Figure 5—source data 6. [file elife-86452-fig5-data6.zip › Fig.5 source data 6.tif]

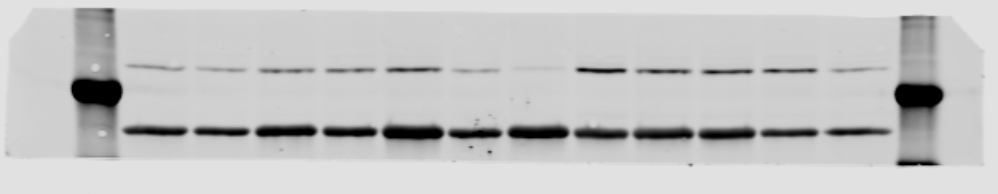

Supplement: Figure 5—source data 7. [file elife-86452-fig5-data7.zip › Fig.5 source data 7.tif]

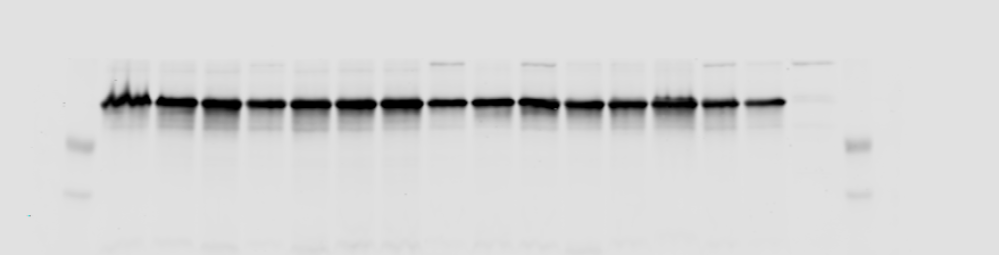

Supplement: Figure 6—source data 2. [file elife-86452-fig6-data2.zip › Fig.6 source data 2.tif]

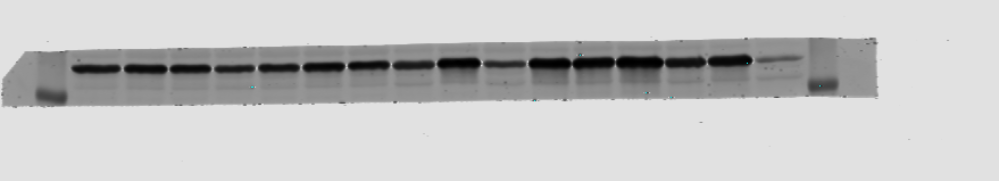

Supplement: Figure 6—source data 3. [file elife-86452-fig6-data3.zip › Fig.6 source data 3.tif]

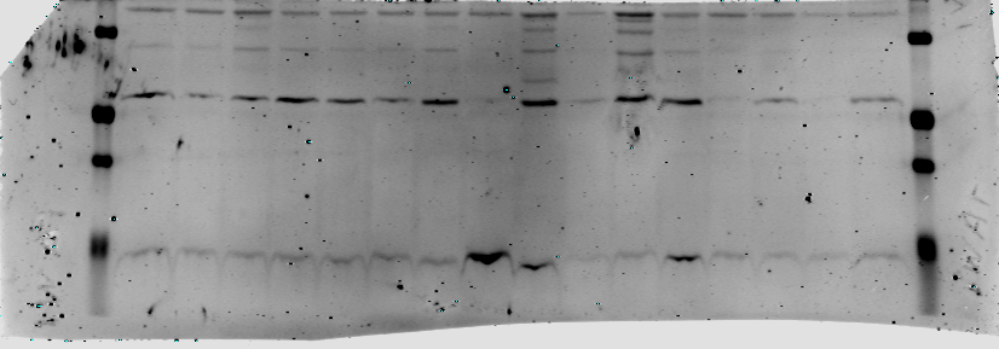

Supplement: Figure 6—source data 4. [file elife-86452-fig6-data4.zip › Fig.6 source data 4.tif]

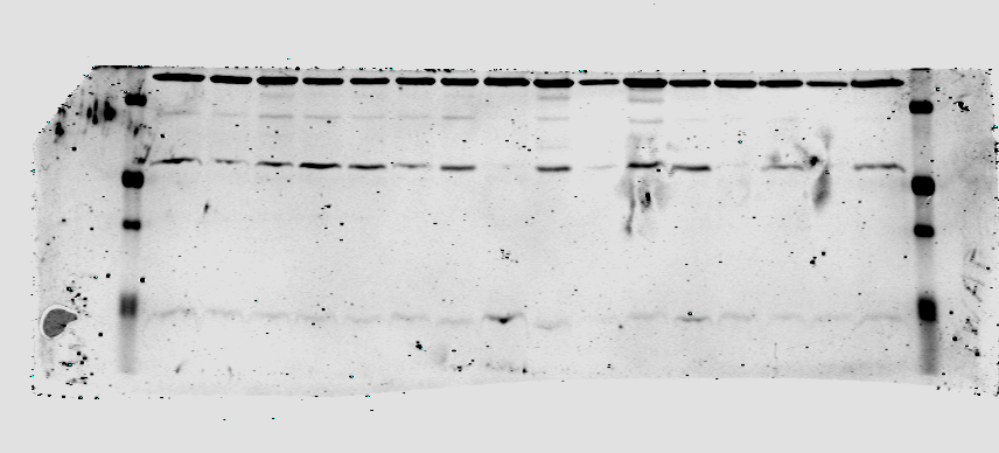

Supplement: Figure 6—source data 5. [file elife-86452-fig6-data5.zip › Fig.6 source data 5.tif]

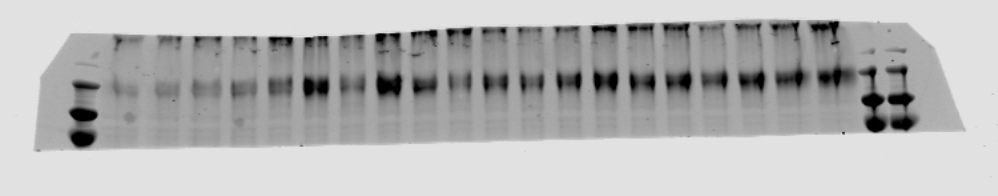

Supplement: Figure 6—source data 6. [file elife-86452-fig6-data6.zip › Fig.6 source data 6.tif]

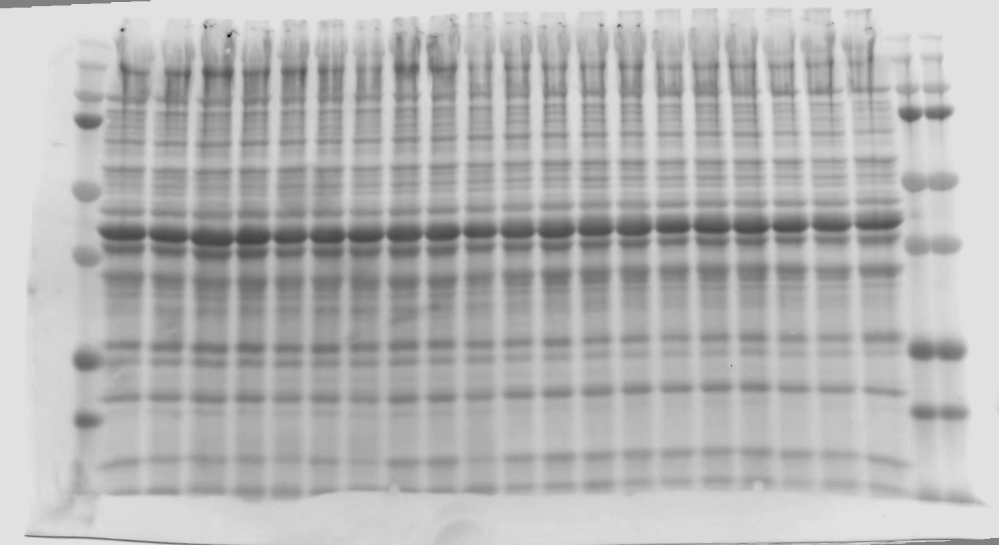

Supplement: Figure 6—source data 7. [file elife-86452-fig6-data7.zip › Fig.6 source data 7.tif]

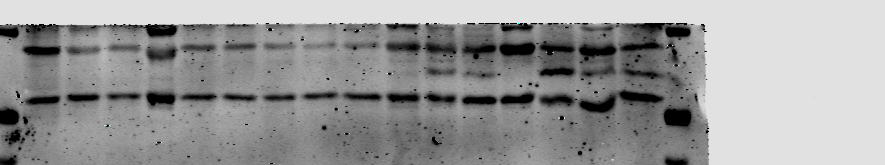

Supplement: Figure 6—figure supplement 1—source data 2. [file elife-86452-fig6-figsupp1-data2.zip › UCP1 scWAT 7-12-18 HFD OPA1 BKO.tiff]

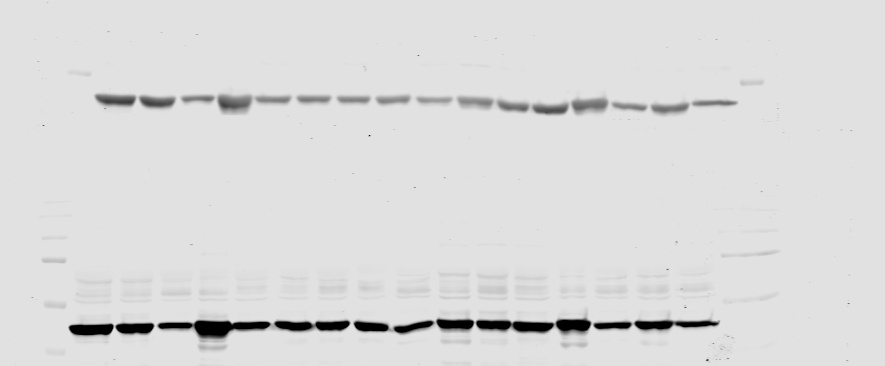

Supplement: Figure 6—figure supplement 1—source data 3. [file elife-86452-fig6-figsupp1-data3.zip › bactin for ucp1 scWAT tissue HFD 7-12-18 copy.tiff]

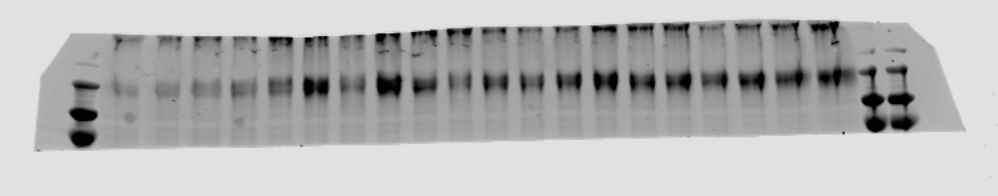

Supplement: Figure 6—figure supplement 1—source data 4. [file elife-86452-fig6-figsupp1-data4.zip › Supplemental Fig.5 source data 4 .tiff]

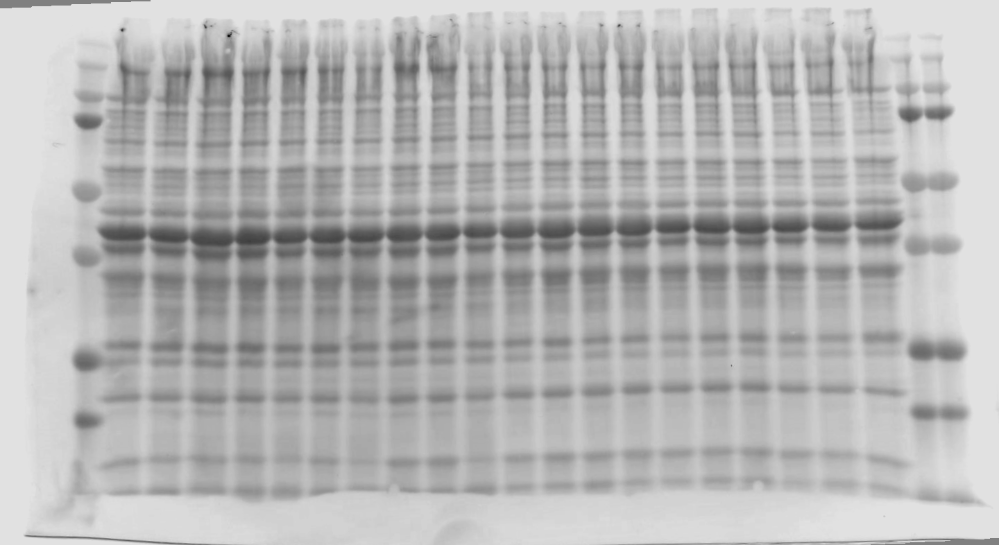

Supplement: Figure 6—figure supplement 1—source data 5. [file elife-86452-fig6-figsupp1-data5.zip › Supplemental Fig.5 source data 5.tiff]

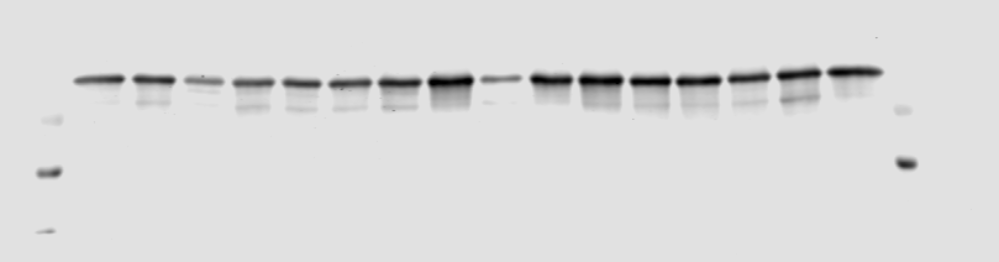

Supplement: Figure 7—source data 2. [file elife-86452-fig7-data2.zip › Fig.7 source data 2.tif]

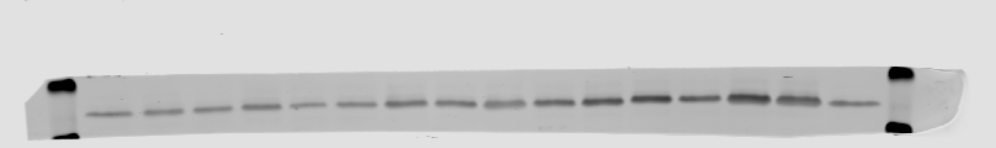

Supplement: Figure 7—source data 3. [file elife-86452-fig7-data3.zip › Fig.7 source data 3.tif]

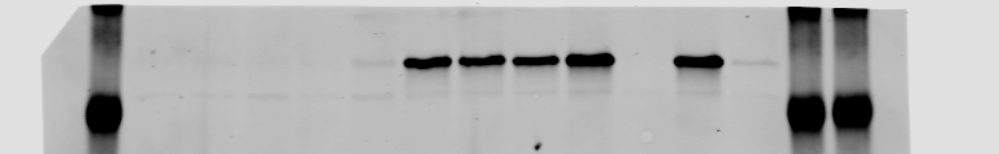

Supplement: Figure 7—source data 4. [file elife-86452-fig7-data4.zip › Fig.7 source data 4.tif]

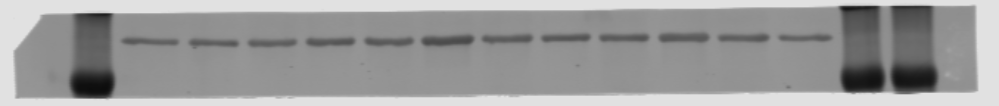

Supplement: Figure 7—source data 5. [file elife-86452-fig7-data5.zip › Fig.7 source data 5.tif]

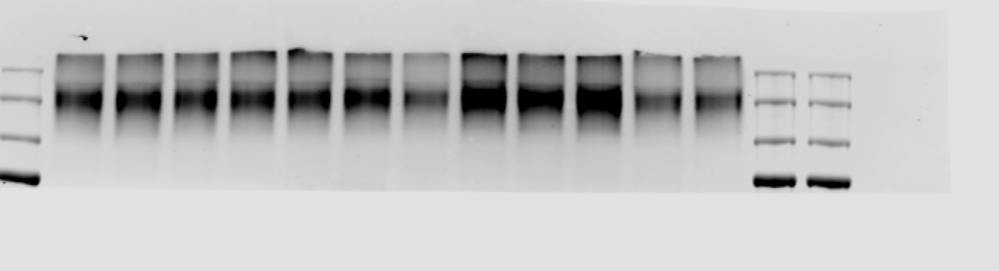

Supplement: Figure 7—source data 6. [file elife-86452-fig7-data6.zip › Fig.7 source data 6.tif]

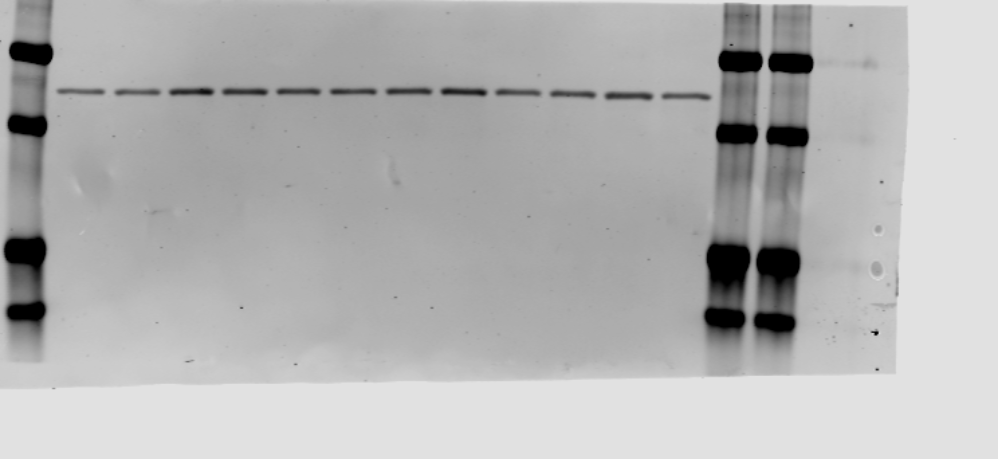

Supplement: Figure 7—source data 7. [file elife-86452-fig7-data7.zip › Fig.7 source data 7.tif]

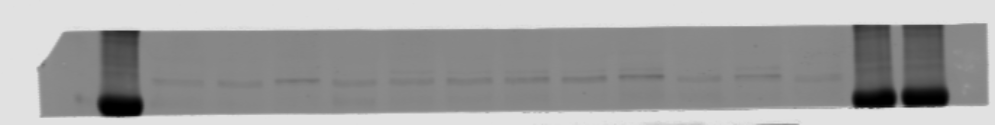

Supplement: Figure 7—figure supplement 1—source data 2. [file elife-86452-fig7-figsupp1-data2.zip › suppl Fig.6 source data 2.tif]

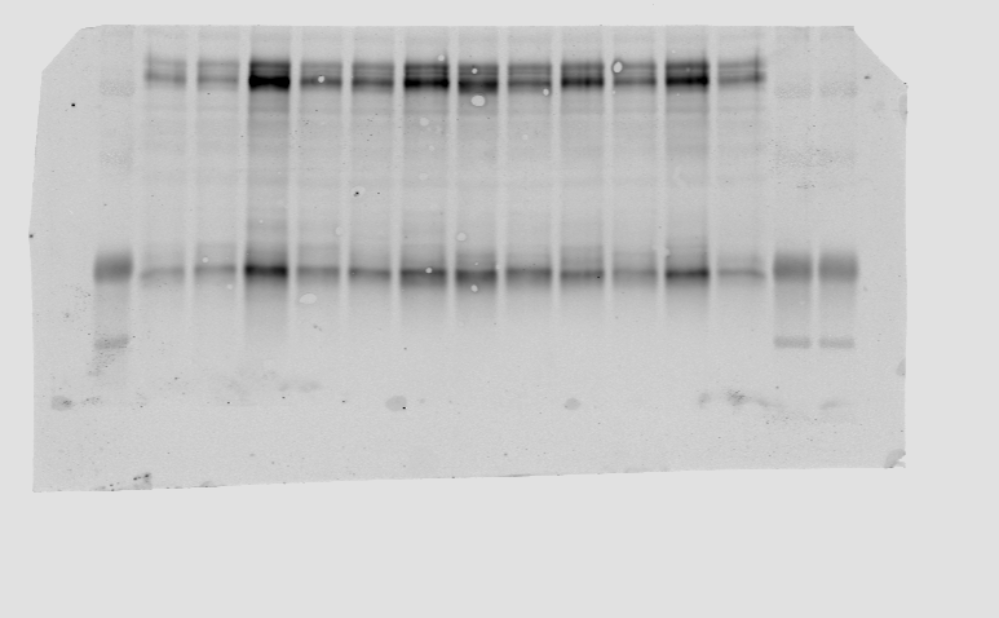

Supplement: Figure 7—figure supplement 1—source data 4. [file elife-86452-fig7-figsupp1-data4.zip › suppl Fig.6 source data 4.tif]

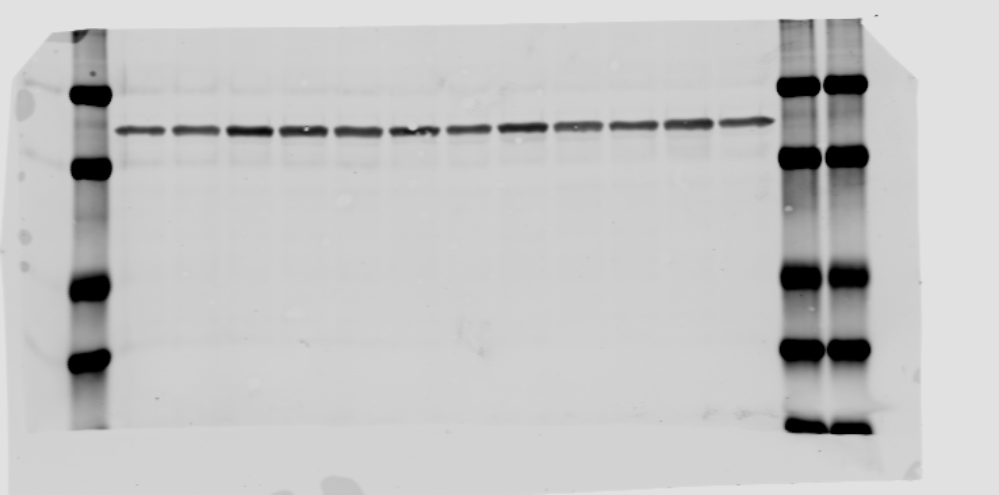

Supplement: Figure 7—figure supplement 1—source data 5. [file elife-86452-fig7-figsupp1-data5.zip › suppl Fig.6 source data 5.tif]
