# Supplementary material for: GDF15 is required for cold-induced thermogenesis and contributes to improved systemic metabolic health following loss of OPA1 in brown adipocytes
Source: eLife. 2023 Oct 11;12:e86452. doi: 10.7554/eLife.86452 (PMC10567111; doi:10.7554/eLife.86452)
Supplement: Figure 5—source data 1. — (G) Full immunoblot images for UCP1 and β-actin in BAT. (M) Full immunoblot images for UCP1 and β-actin in inguinal white adipose tissue (iWAT). (N) Full immunoblot images for tyrosine hydroxylase (TH) and β-actin in iWAT. [file elife-86452-fig5-data1.zip › Fig. 5 - source data 1.pptx]

## Slide 1
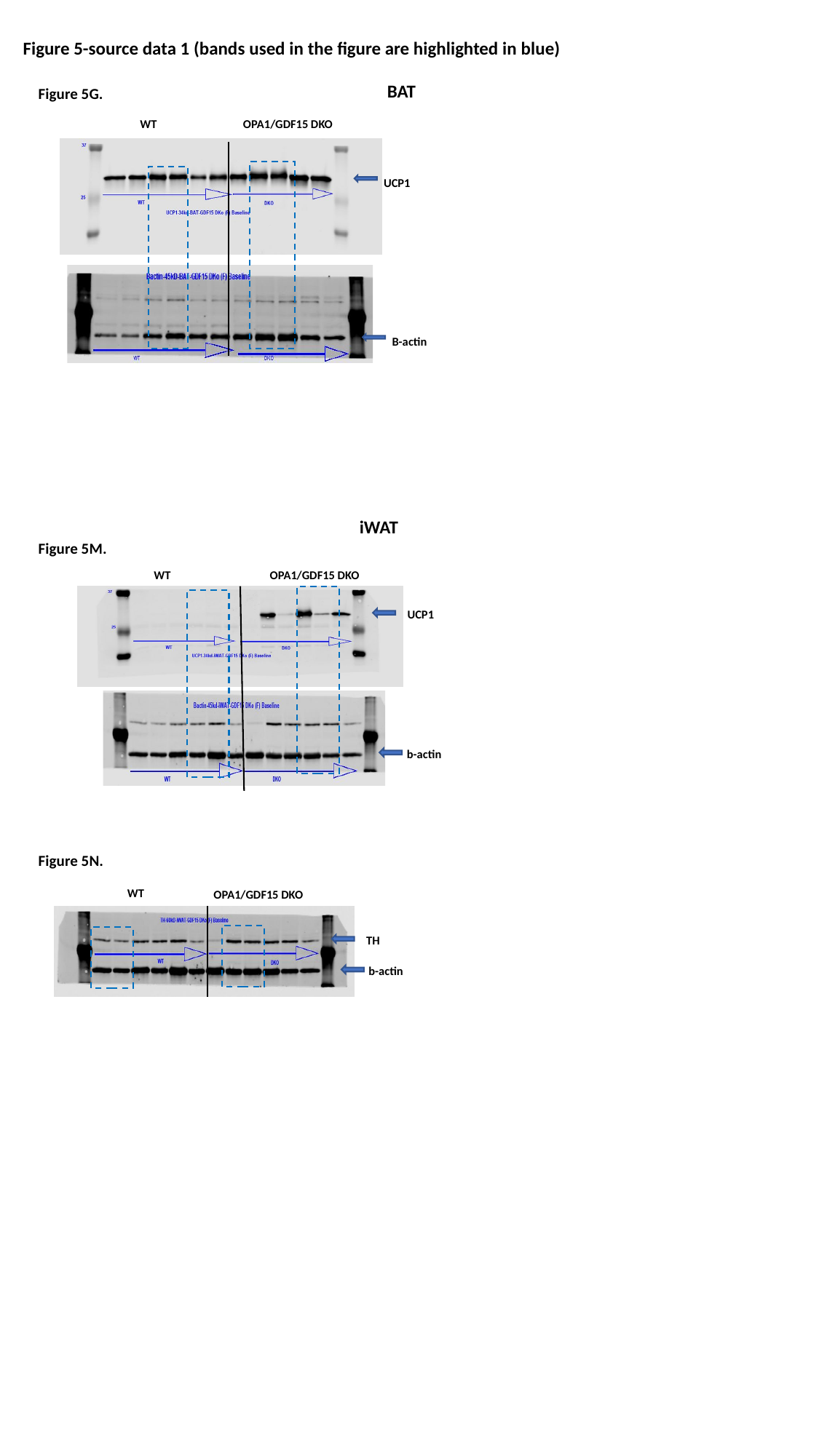

Figure 5-source data 1 (bands used in the figure are highlighted in blue)
BAT
Figure 5G.
WT
OPA1/GDF15 DKO
UCP1
B-actin
c
iWAT
Figure 5M.
WT
OPA1/GDF15 DKO
UCP1
b-actin
Figure 5N.
WT
OPA1/GDF15 DKO
TH
b-actin
