# Supplementary material for: GDF15 is required for cold-induced thermogenesis and contributes to improved systemic metabolic health following loss of OPA1 in brown adipocytes
Source: eLife. 2023 Oct 11;12:e86452. doi: 10.7554/eLife.86452 (PMC10567111; doi:10.7554/eLife.86452)
Supplement: Figure 6—source data 1. — (S) Full immunoblot images for UCP1 and β-actin in brown adipose tissue (BAT). (U) Full immunoblot images for UCP1 and β-actin in inguinal white adipose tissue (iWAT). (V) Full immunoblot for Serca1a in gastrocnemius muscle and Ponceau red staining. [file elife-86452-fig6-data1.zip › Fig. 6 - source data 1.pptx]

## Slide 1
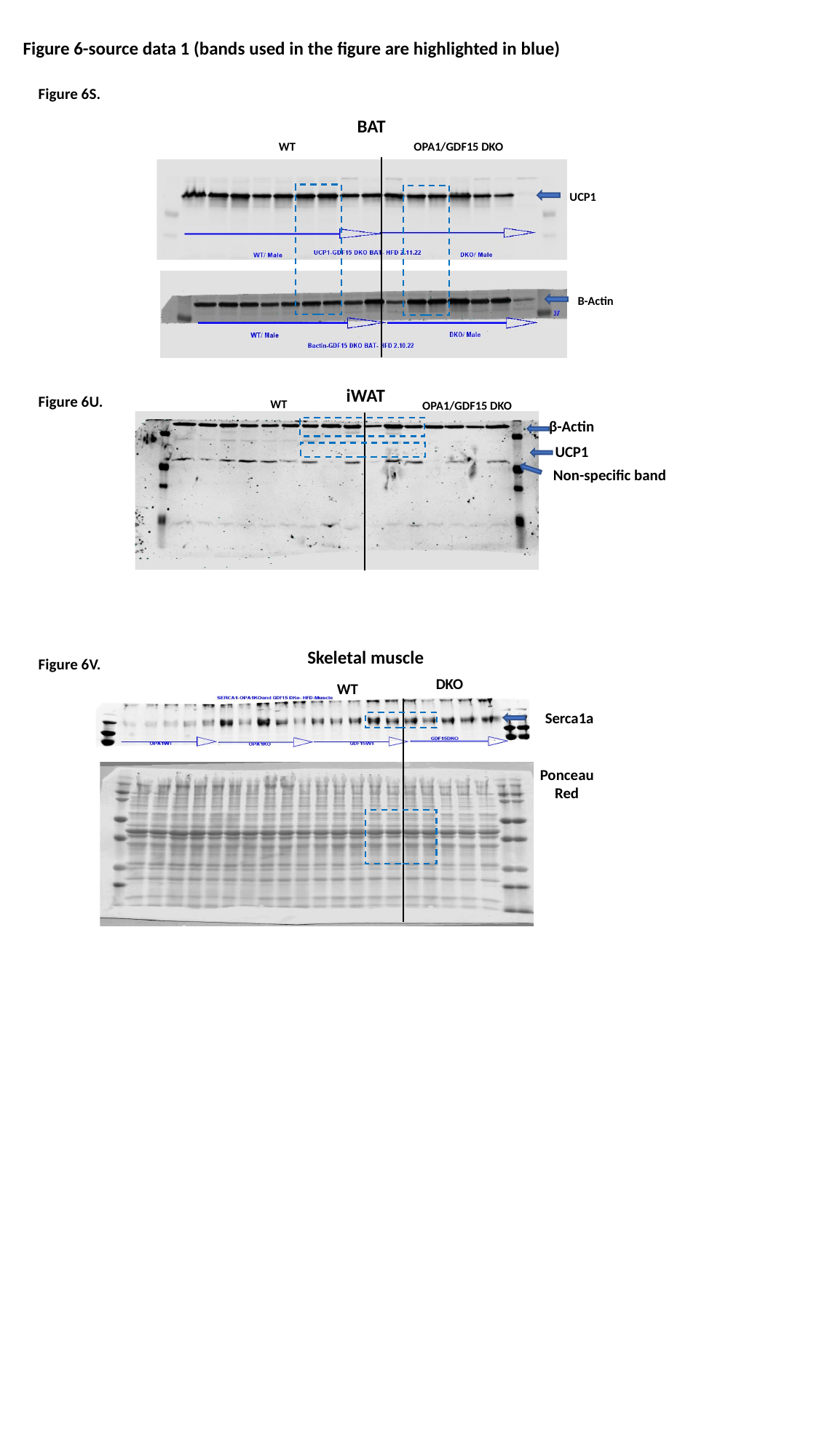

Figure 6-source data 1 (bands used in the figure are highlighted in blue)
Figure 6S.
BAT
WT
OPA1/GDF15 DKO
UCP1
B-Actin
iWAT
Figure 6U.
WT
OPA1/GDF15 DKO
β-Actin
UCP1
Non-specific band
Skeletal muscle
Figure 6V.
DKO
WT
Serca1a
Ponceau Red
