# Supplementary material for: GDF15 is required for cold-induced thermogenesis and contributes to improved systemic metabolic health following loss of OPA1 in brown adipocytes
Source: eLife. 2023 Oct 11;12:e86452. doi: 10.7554/eLife.86452 (PMC10567111; doi:10.7554/eLife.86452)
Supplement: Figure 6—figure supplement 1—source data 1. — (I) Full immunoblot images for UCP1 and β-actin in inguinal white adipose tissue (iWAT) of wild-type (WT) and OPA1 brown adipose tissue (BAT) knockout (KO) mice. (J) Full immunoblot images for Serca1a and Ponceau red staining in gastrocnemius muscle of WT and OPA1 BAT KO mice. [file elife-86452-fig6-figsupp1-data1.zip › Supplemental Fig. 5- source data 1.pptx]

## Slide 1
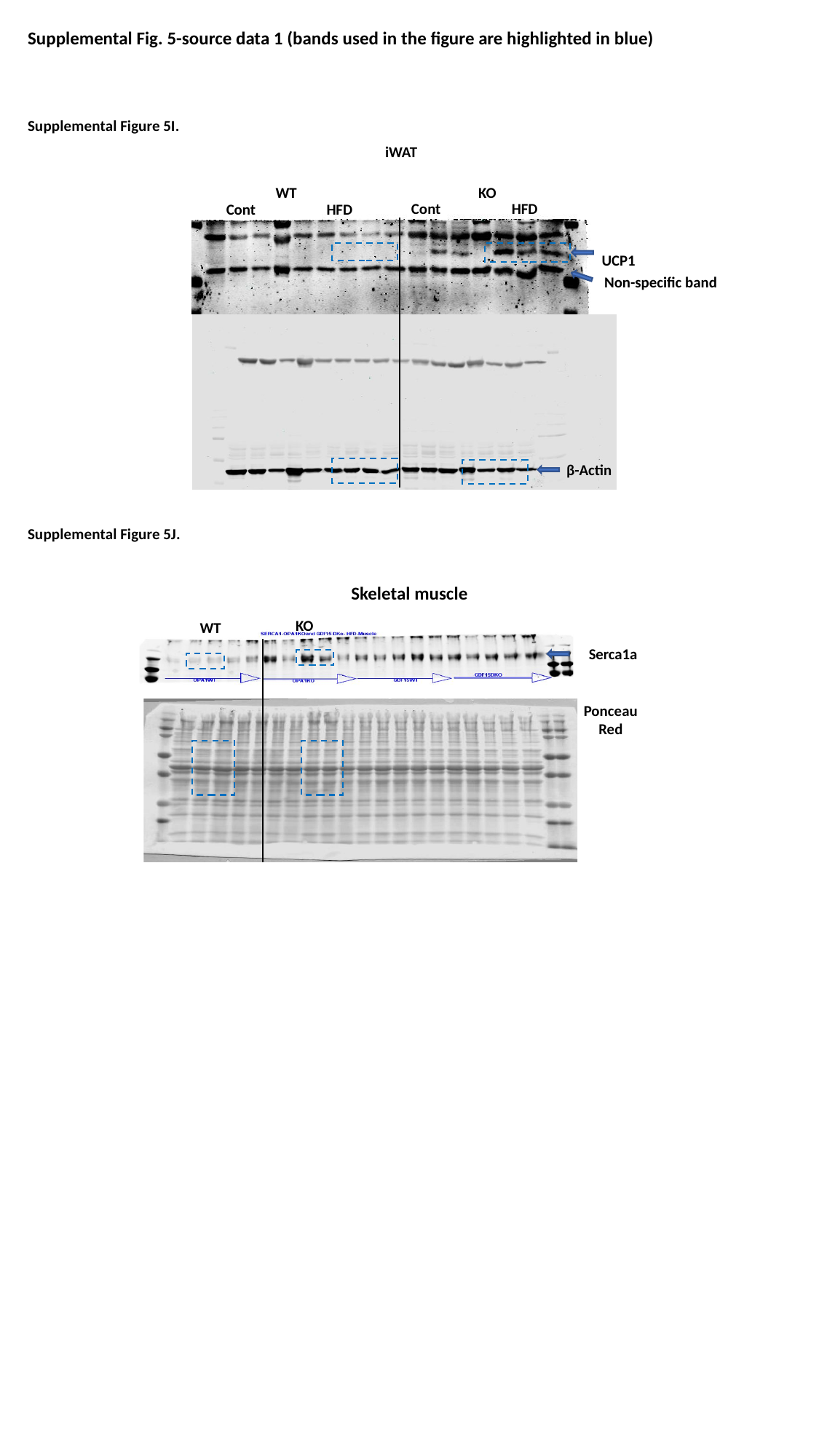

Supplemental Fig. 5-source data 1 (bands used in the figure are highlighted in blue)
Supplemental Figure 5I.
iWAT
WT
KO
UCP1
Cont
HFD
Cont
HFD
Non-specific band
β-Actin
Supplemental Figure 5J.
Skeletal muscle
KO
WT
Serca1a
Ponceau Red
