# Supplementary material for: GDF15 is required for cold-induced thermogenesis and contributes to improved systemic metabolic health following loss of OPA1 in brown adipocytes
Source: eLife. 2023 Oct 11;12:e86452. doi: 10.7554/eLife.86452 (PMC10567111; doi:10.7554/eLife.86452)
Supplement: Figure 7—source data 1. — (G) Full immunoblot images for UCP1 and β-actin in brown adipose tissue (BAT). (M) Full immunoblot images for UCP1 and β-actin in inguinal white adipose tissue (iWAT). (N) Full immunoblot for Serca1a and β-actin in gastrocnemius muscle. [file elife-86452-fig7-data1.zip › Fig. 7 - source data 1.pptx]

## Slide 1
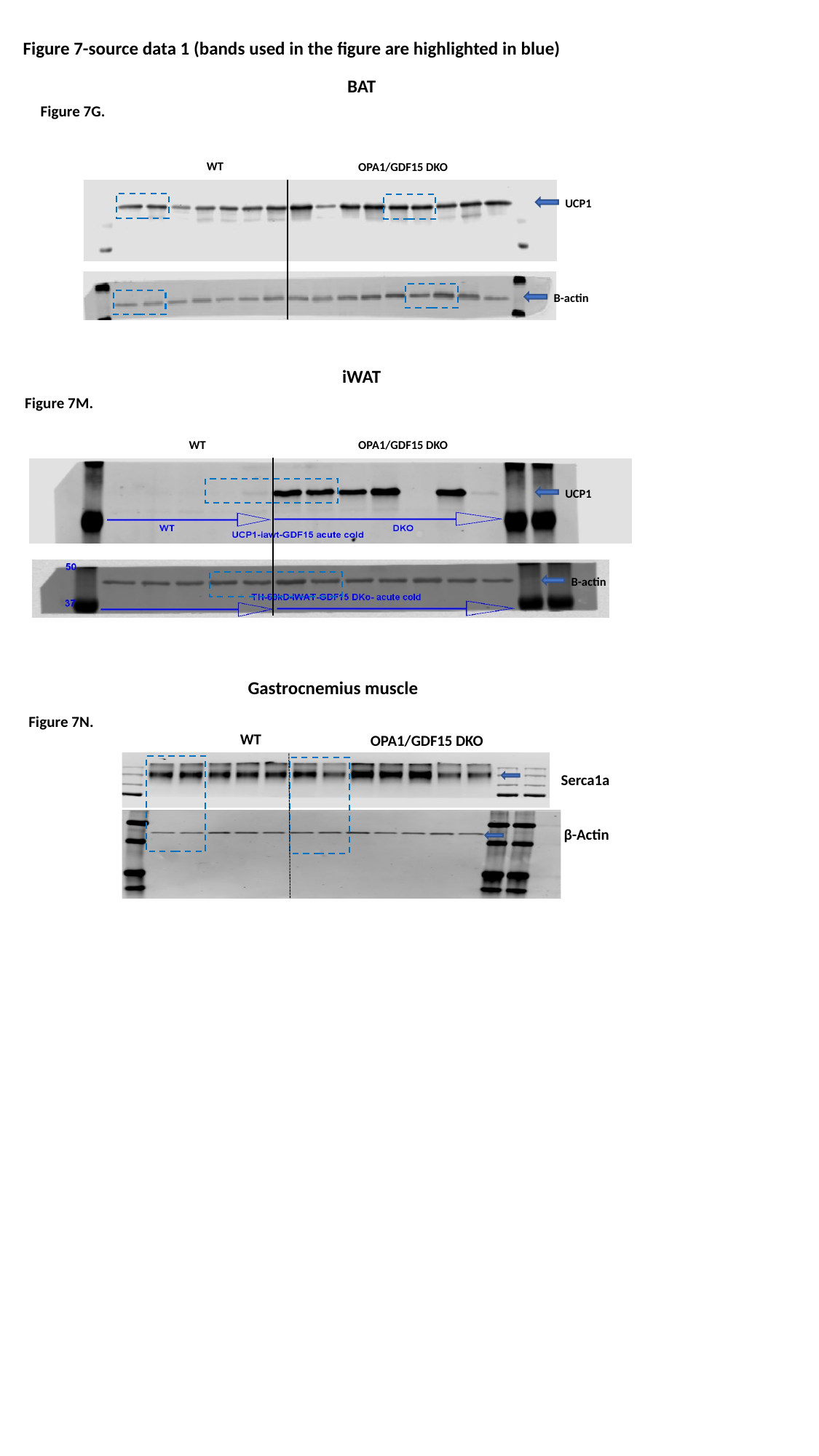

Figure 7-source data 1 (bands used in the figure are highlighted in blue)
BAT
Figure 7G.
WT
OPA1/GDF15 DKO
UCP1
B-actin
iWAT
Figure 7M.
WT
OPA1/GDF15 DKO
UCP1
B-actin
Gastrocnemius muscle
Figure 7N.
WT
OPA1/GDF15 DKO
Serca1a
β-Actin
