# Supplementary material for: GDF15 is required for cold-induced thermogenesis and contributes to improved systemic metabolic health following loss of OPA1 in brown adipocytes
Source: eLife. 2023 Oct 11;12:e86452. doi: 10.7554/eLife.86452 (PMC10567111; doi:10.7554/eLife.86452)
Supplement: Figure 7—figure supplement 1—source data 1. — (J) Full immunoblot images for GLUT1 and β-actin in skeletal muscle of WT and OPA1/GDF15 BAT DKO mice. [file elife-86452-fig7-figsupp1-data1.zip › Supplemental Fig. 6 - source data 1.pptx]

## Slide 1
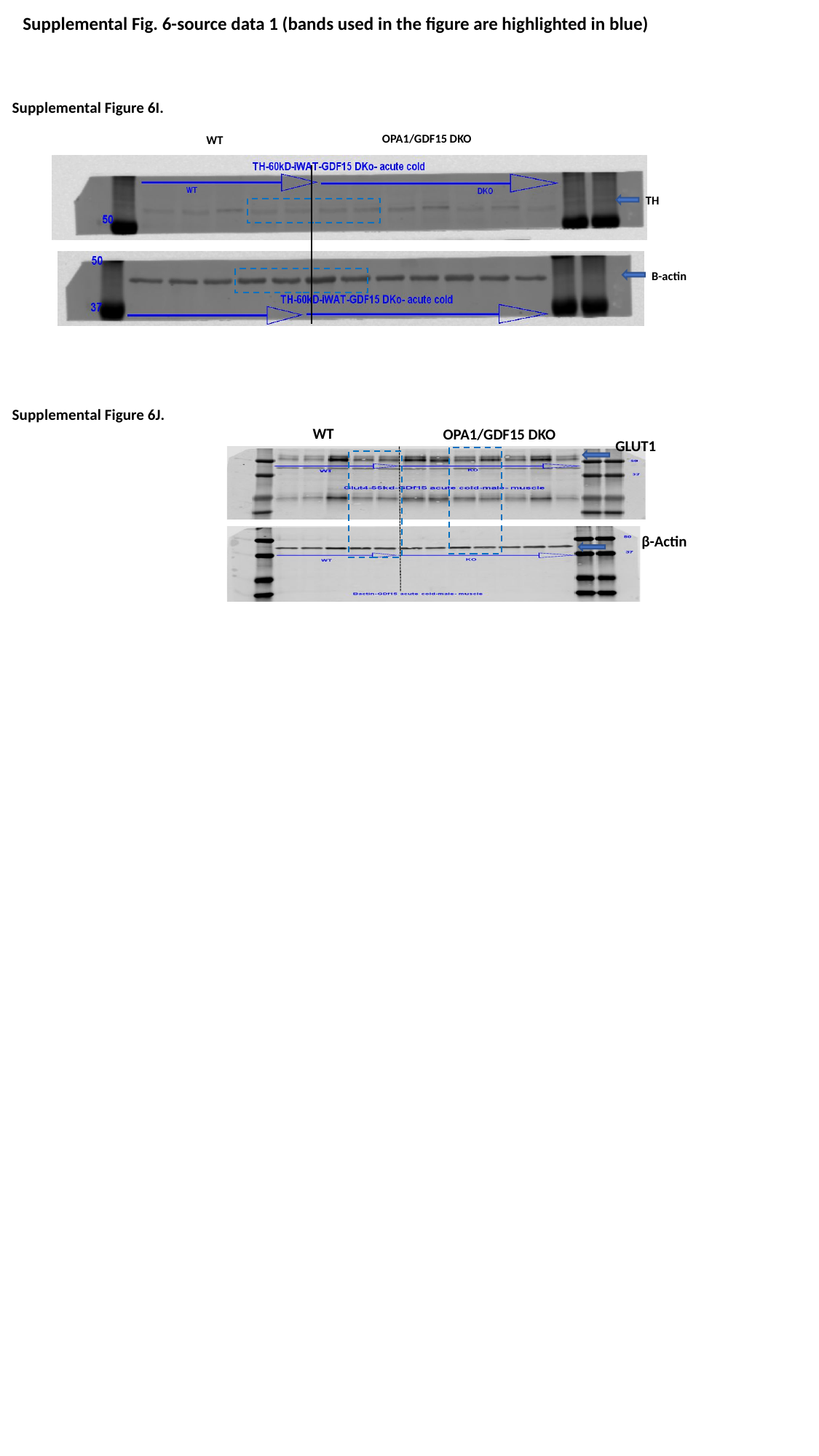

Supplemental Fig. 6-source data 1 (bands used in the figure are highlighted in blue)
Supplemental Figure 6I.
OPA1/GDF15 DKO
WT
TH
B-actin
Supplemental Figure 6J.
WT
OPA1/GDF15 DKO
GLUT1
β-Actin
